# Exergames to Limit Weight Gain and to Fight Sedentarism in Children and Adolescents with Obesity

**DOI:** 10.3390/children10060928

**Published:** 2023-05-24

**Authors:** Valeria Calcaterra, Matteo Vandoni, Luca Marin, Vittoria Carnevale Pellino, Virginia Rossi, Alessandro Gatti, Pamela Patanè, Caterina Cavallo, Fabio Re, Ilaria Albanese, Dario Silvestri, Alessandro De Nunzio, Gianvincenzo Zuccotti

**Affiliations:** 1 Department of Internal Medicine and Therapeutics, University of Pavia, 27100 Pavia, Italy; valeria.calcaterra@unipv.it; 2 Department of Pediatrics, Vittore Buzzi Children’s Hospital, 20154 Milan, Italy; virginia.rossi@unimi.it (V.R.); gianvincenzo.zuccotti@unimi.it (G.Z.); 3 Laboratory of Adapted Motor Activity (LAMA), Department of Public Health, Experimental Medicine and Forensic Science, University of Pavia, 27100 Pavia, Italy; matteo.vandoni@unipv.it (M.V.); vittoria.carnevalepellino@unipv.it (V.C.P.); alessandro.gatti08@universitadipavia.it (A.G.); pamela.patane01@universitadipavia.it (P.P.); cavallo.caterina@stud.lunex-university.net (C.C.); ilaria.albanese01@universitadipavia.it (I.A.); 4 Department of Physiotherapy, Faculty of Medicine, University of Ostrava, 703 00 Ostrava, Czech Republic; 5 Laboratory for Rehabilitation, Medicine and Sport (LARMS), 00133 Rome, Italy; fabio.re01@universitadipavia.it; 6 Department of Industrial Engineering, University of Rome Tor Vergata, 00133 Rome, Italy; 7 Department of Research and Development, LUNEX International University of Health, Exercise and Sports, Avenue du Parc des Sports, 50, 4671 Differdange, Luxembourg; alessandro.denunzio@lunex-university.net; 8 Department of Research, ASOMI College of Sciences, 2080 Marsa, Malta; dario.silvestri@acs-college.com; 9 Department of Biomedical and Clinical Sciences, Università di Milano, 20122 Milan, Italy

**Keywords:** exergames, active videogames, weight, sedentarism, children, adolescents, obesity

## Abstract

Exergames are defined as digital games that require bodily movements to play, stimulating an active gaming experience to function as a form of physical activity (PA). The players interact with the game through whole-body movements improving energy expenditure. Exergames may be effective in improving physical and psychological aspects of children and adolescents with obesity. In this narrative review, we synthesized the current evidence regarding the role of exergames in modifying body composition and weight and in promoting changes in sedentary behavior to define the benefits of active video games as useful tools for fighting sedentarism and to outline the future directions of exergaming as a supplementation exercise rather than a replacement in educational programs for pediatric obesity. Data from the literature indicate that exergames may offer an interesting impact on childhood obesity and may be considered a potential strategy for controlling weight gain and body composition, promote PA, and decrease time spent on sedentary behavior in children and adolescents with obesity. However, exergame use also has some limits, such as children’s poor self-regulation and poor structuring of exergame use. Therefore, a prudent approach should be maintained, and additional high-quality research is needed to determine if exergames can be effectively used in the treatment of childhood obesity and if new digital media, as a supplementation of exercise rather than a replacement, could be considered to combat sedentary behavior in educational programs for pediatric obesity prevention.

## 1. Introduction

The global prevalence of pediatric obesity (OB) is rising and thus emerging as a global public health problem [1,2].

Childhood obesity is a multisystem condition that has various comorbidities, such as cardiometabolic and respiratory disorders, endocrinological problems, gastrointestinal diseases, orthopedic disorders, and psychological problems; OB also affects the function of the immune system, leading to systemic low-grade chronic inflammation [3]. All these harmful OB-related consequences contribute to premature mortality in adulthood [4].

Promoting PA and an active lifestyle is considered an effective approach to treating obesity in children because it helps manage weight and it produces physical and mental health benefits, representing a nonpharmacological approach to attenuating complications related to obesity [5].

OB is caused by an energy imbalance that results from increased food intake, unhealthy food choices, and inadequate physical activity (PA) [6]. Different factors may contribute to decreased PA levels, including the increased sedentary time in front of the screen [6]. Thus, recently, exergames (also referred to as active videogames) have emerged as an innovative approach to managing sedentary behavior and health in children and adolescents [2,7]. Exergames are defined as digital games that require bodily movements to play, stimulating an active gaming experience to function as a form of PA [2,7,8]. The players interact with the game through whole-body, movements improving energy expenditure [9,10]. Exergames may be effective in improving physical and psychological aspects of children and adolescents with OB [2,11].

In this narrative review, we synthesized the current evidence regarding the role of exergames in modifying body composition and weight and promoting changes in sedentary behavior in order to define the benefits of active video games as useful tools for fighting sedentarism and to outline the future directions of exergaming as supplementation of exercise rather than a replacement in educational programs for pediatric obesity.

## 2. Materials and Methods

**Figure 1:**
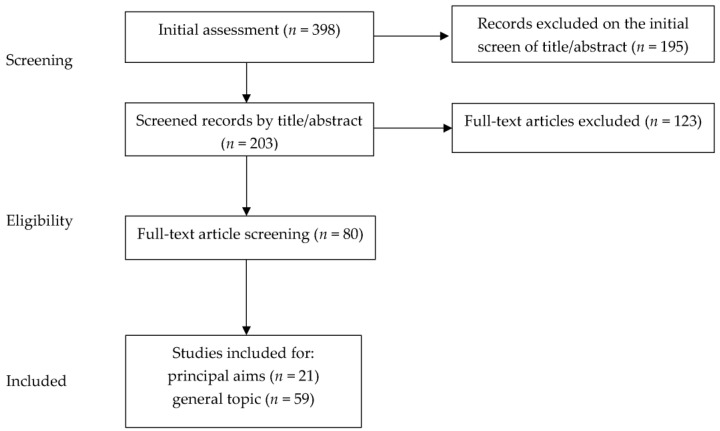
Flowchart of criteria for study selection.

We conducted a literature narrative review [12,13] on the topic of the role of exergames in body composition and weight modifications and changes in sedentary behavior. We included English language articles, including original studies, guidelines, consensus position statements and commentaries, reviews, and meta-analyses published in the last 15 years up to April 2023. Case reports and case series were excluded. Starting from a total of 398 papers, the authors assessed the abstracts (*n* = 203), screened the full texts of potentially relevant articles, and reviewed the full texts of relevant articles (*n* = 80). In Figure 1, the diagram of the selection method is reported. The characteristics of the selected relevant studies in analyzing specific aims, such as the role of exergames in improving body composition and reducing body weight and identifying the effectiveness of exergames in limiting sedentary behavior, are also reported in Table 1, Table 2, Table 3 and Table 4. The reference list of all articles was also checked to identify additional relevant studies. Regarding search terms, alone and/or in combination, we used the following: physical activity, exergames, exergaming, weight, body composition, sedentary behavior, obesity, adolescents, children, and sedentarism. PubMed, Scopus, and Web of Science were used as platforms for the research.

## 3. Physical Activity in Children and Adolescents with Obesity

Regular physical activity (PA) practice is crucial for promoting a healthy lifestyle, especially for children and adolescents with OB [3,33,34]. PA refers to any body movement that requires energy expenditure and is produced by skeletal muscles and includes different types of activities, such as aerobic, resistance, and flexibility activities [33]. Recently, several studies have shown that PA plays a crucial role in the management of OB in children and adolescents. In fact, PA can help to reduce body weight, improve body composition, and decrease insulin resistance, as well as strengthen self-esteem and improve the overall quality of life in this population [34,35,36,37,38]. Moreover, positive effects on mental health, including reducing symptoms of anxiety and depression [39], body mass index (BMI), BMI z-score, and body fat percentage in children and adolescents with OB have been associated with PA practice [40,41,42], with positive effects on the severity of OB and future adverse complications also being observed. Finally, PA plays a crucial role in promoting physical fitness in children and adolescents with OB in terms of cardiovascular fitness, muscle strength, and endurance enhancements with a consequent improvement of health status [40,43,44].

In recent years, numerous studies have examined the effectiveness of different types of exercise training. While some studies in the past have suggested that aerobic training is superior to resistance training and other types of training in improving health in children with OB, current research indicates that a combination of aerobic and resistance training, known as concurrent training, is the most effective method for improving health and reducing OB in children [35,45]. Despite the benefits of practicing PA, more than about 75% of children and adolescents with OB are inactive [46] and do not reach 60 min per day or 7 h per week doing PA [47] mainly due to barriers in participating in regular exercise [48]. These barriers include limited access to safe and affordable recreational spaces, a lack of resources, and self-consciousness or embarrassment of body size. Key features in contrasting this phenomenon are the improvement of physical fitness and increased enjoyment during PA practice. In fact, as shown by Stodden et al. [49] and lately confirmed by Harter et al. [50], improving physical fitness and motor competence in children and adolescents with OB increases their self-competence and consequently enhances the adherence to a PA program, creating a positive spiral of engagement, improving health and reducing fat mass. Furthermore, enjoyable and engaging PA programs can help children to improve physical fitness and increase motivation and adherence to regular exercise in this population [38,50,51]. For example, technology-based interventions, such as activity trackers, mobile applications, and exergames, have been shown to be effective in increasing PA [30,32,52,53]. For these reasons, it is crucial to design PA programs that are enjoyable and engaging to ensure long-term adherence to regular exercise among children and adolescents with OB. These actions could help the promotion of a healthy and active lifestyle, the amelioration of physical and mental health outcomes, and the prevention of the onset of OB-related health conditions.

## 4. Exergames

Exergames combine PA with advanced technology into digital games, which entail body movement as part of the gameplay. The founding principle of exercise gamification lies on encouraging people to engage in physical activity by essentially playing a video game [54,55]. Depending on the training goal, as well as the subject’s health status, there is an extensive variety of gamified exercise for upper and/or lower limb movements that may additionally involve cognitive tasks [54,56].

Exergames are generally based on biofeedback systems, which involve the use of sensors that are attached to the participant’s body and transmit information about specific body parameters (e.g., limb position, muscular contractions, plantar pressure), creating a dynamic interaction between the participant and the game [57].

As mentioned above, literature shows that exergames are an effective strategy to promoting PA and improving adherence to training programs in both young and elderly populations [58].

Indeed, they are used in pediatric rehabilitation settings for various clinical conditions but also as part of post-traumatic event treatment (e.g., injuries) [58,59].

In particular, the introduction of exergames to training protocols have been shown to lead to a greater involvement in physical exercise treatment protocols, facilitating the adoption of healthier behaviors and the development of coordinative abilities in clinical child populations [55,60]. Indeed, a higher level of motor coordination is associated with a reduced risk of adopting an inactive lifestyle, simultaneously reducing the probability of developing negative health-related consequences associated with sedentary behavior [58]. Moreover, past studies showed that exergaming approaches are an effective tool to promoting physical activities and tackling sedentariness, thus leading to positive health-related outcomes even in overweight or obese pediatric populations [54,56,61]. The use of exergames seems particularly successful with very young subjects due to the relatability and similarity to what children live and do in their everyday life. In fact, nowadays it is becoming usual for children to have their personal smartphone or for them to be allowed to use their parents’, where they can easily download different videogames. Therefore, exergames may still catch children’s interest, as classical videogames do, while also inspiring them to perform physical activity [56].

Overall, exergames can be a valuable tool for pediatric healthcare providers and can have a positive impact on the health and well-being of children.

## 5. Exergames in Childhood Obesity

### 5.1. The Role of Exergames in Modifying Body Composition and Weight

Physical inactivity represent a major health burden, comparable to obesity and smoking, contributing to more than 5.3 million deaths worldwide [1,62]. In fact, physical inactivity is a key factor in the increase of fat storage and adiposity [63,64], so practicing physical activity and combating sedentariness are known as promising strategies for preventing childhood obesity [65,66]. Globally, children and young people are becoming less active [1,67] and increasing their time spent in sedentary activities, such as watching television and playing videogames. The recommended daily physical activity levels for children are at least 1 h of moderate-to-vigorous-intensity physical activity [63,68] and less than 2 h of time spent on the screen [63,69]. However, only 1 in 5 children meet the physical activity recommendations, and about 1 in 3 children meet the screen time guidelines [70].

Mass interest in video games among children has brought forth a new technology: exergaming, also known as active video games [63]. Exergames have emerged as an innovative approach to combating childhood obesity [1,2]. Exergames capitalize on children’s interest in computerized video games and their need for increased physical activity [71]. In fact, a variety of body movements such as jumping, kicking, punching, and dodging are set in motion through exergames [1]. As several studies have shown, exergames can generate a variety of physical (e.g., improved fitness, weight loss), psychological (e.g., increased enjoyment), and cognitive benefits among children and adolescents [2,10,72,73,74].

Despite the fact that exergaming consoles are often thought of as home entertainment used by children and adolescents, it is actually in schools where exergames are used as a means of preventing childhood obesity and associated issues [2]. Notably, most of the studies related to exergames have been conducted in U.S. schools in the last decade, perhaps even secondary to the epidemic of childhood obesity in the United States over the past decade [2].

Most exergames appear to increase children’s exercise intensity to a moderate level of physical activity intensity; in fact, in terms of energy expenditure, heart rate, and perceived exertion, exergames effects are comparable to those of walking at moderate intensity (5.7 km/h) in normal weight children (10–13 years old) [2,9]. However, the effects of physical activity achieved with exergames depend on the physical exertion that the games themselves require [2,75]. In particular, games that involve lower limb muscle groups are more demanding since they involve greater muscle mass and thus result in greater energy expenditure than do those that engage only the upper body [2,75]. For instance, the most active exergames are, e.g., dance simulation products (e.g., DDR Dance Dance Revolution^®^) and Wii boxing^®^, the practice of which should be encouraged in order to fight pediatric obesity or prevent it [2]. Some researchers have shown that physical activity performed through exergames exerts significantly positive effects in countering or preventing pediatric obesity, acting both in terms of body composition and cardiovascular fitness [2,52]. DDR^®^ is one of the exergames used as an alternative to sedentary behaviors [75]. Bethea and colleagues [14] observed how DDR^®^ can be particularly useful, either at home or at school, for exercising. They observed an improvement in physical fitness in the first 12 weeks of DDR^®^ training, with gains maintained up to 30 weeks, including in cardiovascular fitness. Other previous studies had already shown that DDR is comparable to other forms of moderate-to-vigorous PA, such as playing tennis and moderate-intensity walking [9], and that following DDR^®^ training, overweight youth improve their fitness [14]. Similarly, in the study by Murphy et al. [15] overweight children who practiced DDR had a decrease in body weight, as well as a significant increase in total exercise time and VO2 max (maximal oxygen consumption) compared with control children.

Other researchers have also shown how exergames (e.g., Eyetoy, Kinect Sport) produce a positive effect on physical activity levels, even leading to a reduction in BMI and body fat [16,19,29] compared with control children. For example, Ni Mhurchu et al. [16] conducted a 12-week pediatric-based randomized pilot study, demonstrating how active video games can be an effective means of increasing children’s overall physical activity levels and improving fitness. In fact, children who played exergames achieved a reduction in waist circumferences compared with controls.

Considering studies conducted specifically on pediatric and adolescent populations with overweight and/or obesity, Zeng and Gao’s review [76] showed how four out of seven RCTs using adiposity as an outcome variable showed significant improvement in BMI, body composition, or body fat, while the other three did not observe significant changes. In particular, Staiano et al. [11] demonstrated weight loss from exergame play in overweight or obese African American adolescents ages 15–19 years. An RCT by Staiano et al. [17] showed that practicing dance-based exergames provides sufficient aerobic intensity to reduce adiposity and increase bone mineral density {BMD} in overweight and obese girls aged 14 to 18 years. Trost et al. [18] showed that playing exergames had favorable results in terms of weight reduction and BMI z-score, as well as in terms of physical activity and fitness level. Meanwhile, Maddison et al. [19] conducted a study to evaluate the effectiveness of using active video games at home and observed that these were able to produce a reduction in BMI and in fat percentage; in contrast, the study by Trost et al. [18], however, observed no difference in the change of physical activities measured with the accelerometer or in physical fitness.

Martínez-López et al. [22] conducted an 8-week longitudinal quantitative study of adolescents aged 12 to 15 years, evaluating the effects of playing Pokémon GO. This augmented reality game (ARG) is based on leveling up with Pokémon by performing different tasks and movements in a variety of physical locations with the aid of cell phone GPS [77]. Therefore, Pokémon GO promotes motivation to play a video game while increasing daily PA levels [78]. In the study by Martínez-López et al. [22], participants increased their PA levels and cardiorespiratory fitness (CRF), while BMI was reduced in adolescents. However, the parameters of speed/agility (S/A), muscular strength (MS), and waist–hip index (WHI) did not change with this ARG. Notably, inactive students showed more significant improvements in CRF and percent fat mass (%BF) than did other participants regardless of age, gender, number of computers, and maternal education. In addition, most of the players felt that playing Pokémon GO improved their health and was a good way to lose weight [22].

However, the literature is not unanimous regarding exergames’ effects on pediatric fitness level [76]. According to some studies, such as that of Baranowski et al. [20], activity performed with exergames exerts no effect on children’s body composition (body mass index and body fat percentage) or PA levels [2,20].

Moreover, a limitation for the effectiveness of exergames in pediatric age depends on their poorly structured use, characterized by poor self-regulation [2]. In fact, children are not as rational as adults in regulating their thoughts and behaviors while playing exergames and do not think about the potential purposes of practicing them [2]. However, Gao et al. [21] suggested children to set goals in exergame play; doing so, they observed that children who set specific attainable goals had better health outcomes than did those who set vague, “do your best” goals. These goals in fact provide children with a focus of direction and the subsequent generation of clear behavioral intentions.

In addition, unlike traditional exercise, exergaming, as documented by Jakobsson [79], is capable of stimulating feelings of pleasure through virtual rewards, which lead to feelings of accomplishment and thus may contribute to greater adherence than in a traditional PA program [79]. Likewise, in another study performed in Canada that compared interactive video games with traditional aerobic training, participants in the exergaming group attended 30 percent more than did participants in the traditional training group [80]. As a result, one of the most appealing aspects of exergames lies in their enjoyment and motivation [2]. Most exergames, besides requiring physical effort, are perceived as fun by children and adolescents [2].

Regarding gender differences observed in the assessed studies, Murphy et al. [15] observed that girls engaging in physical activity experienced a significantly greater reduction in systolic blood pressure than did boys. In addition, the female intervention group, besides gaining significantly less weight than the control group, showed a reduction in IL-6 levels and an increase in relative peak VO2 (peak oxygen uptake), while the control group showed the opposite trend. In males, the only significant difference between the intervention and control groups was observed for l-arginine, which was used as a marker of NO production and was significantly decreased in the intervention group. Ni Mhurchu et al.’s study [16] revealed that boys were more active than were girls when activity times were combined (light, moderate, and vigorous). Gao et al.’s research [21] showed that boys had higher levels of physical activity than did girls, but both sexes had similar DDR performance scores. Martínez-López et al.’s study [22] found that boys achieved higher levels of play, accumulated more points, and caught more Pokémon than did girls. Boys also expressed a greater belief that the augmented reality game was an effective way to lose weight and showed a greater willingness to try new versions of the game. Bethea et al. [14], Staiano et al. [11], Trost et al. [18], Maddison et al. [19], and Baranowski et al. [20] found no significant differences between genders in their studies. Finally, another study by Staiano et al. [17] involved only girls. These findings point to several differences and similarities between the sexes in terms of physical activity levels, health outcomes, and attitudes toward exergames.

In Table 1 and Table 2, the main characteristics of the investigated studies and the investigated outcomes and the related tools used are summarized.

It is interesting to compare the affordability of exergames and AVR in different areas of the world. In developed and technologically advanced countries, access to exergames is widespread due to the greater availability of game consoles, personal computers, smartphones, and high-speed Internet connections. In contrast, in economically and technologically underdeveloped countries or regions, access to AVRs may be limited, leading to lower participation in exergames and greater dependence on traditional physical activities. However, the availability and affordability of exergame technologies may also vary within countries or regions, between city and country, and across different socioeconomic levels. Scientific studies addressing exergames use between technologically advanced countries and those with poor economic and technological development in children and adolescents with obesity are not yet currently available. However, Smits-Engelsman et al. [81] conducted a trial involving children with developmental coordination disorder attending a primary school in a low-income community in South Africa. Through a 5-week training program using Wii Fit games, Smits-Engelsman et al. [81] observed how active play improved functional strength and anaerobic fitness in both the intervention group and control group composed of typically developing peers.

Overall, it has been observed that exergames can provide immediate benefits. However, it is important to evaluate whether the positive effects produced by exergames are long-lasting. Some studies have suggested that exergames may have difficulty maintaining their effects over time due to factors such as decreased motivation, boredom, and lack of variety in play [82,83].

According to Amy Shirong Lu et al. [84], enriching exergames with stories could facilitate players’ compliance to play them, keeping involvement in the game high. Indeed, these are supposed to facilitate players’ immersion process, as demonstrated in the study that evaluated the game Escape from Diab [85]. Notably, the latter study also documented improved immersion effects and several health outcomes in the case of ethnic similarity between video game characters and players [23]. Narrative exergames, which combine storytelling elements with physical activity, are promising but face several challenges. Primarily, integrating narratives with gameplay and creating the game within the narrative can be difficult [84]. Creating games that are inherently narrative and physically active is a potential solution. However, many current narrative games focus on a single protagonist, limiting player identification and involvement. Multicharacter video games appear to be the most engaging; however, they are also challenging in terms of design and cost [84]. The limited ability to process information during exergames is another challenge. Players must coordinate hand–eye–body movements, leading to intense physical exertion and affecting players’ ability to engage with narratives in exergames [84]. A further challenge is the effect of narrative saturation, whereby excessive immersion in a story can lead to negative consequences in the real world [84]. For example, players may develop unrealistic expectations about the effects of exercise after following a poignant narrative. Eventually, understanding player interaction with game characters in exergames is still an open challenge that needs further investigation [84].

The limited number of long-term studies on exergames presents a challenge when trying to assess their lasting impact. However, long-term studies would provide a better understanding of the sustainability of the effects of exergames and identify strategies for optimizing their impact. Further research in this area is critical for a complete understanding of interventions with exergames and their potential as a sustainable means of promoting physical activity and health.

### 5.2. Use of Exergames to Reduce Time Spent in Sedentary Behavior

**Figure 2:**
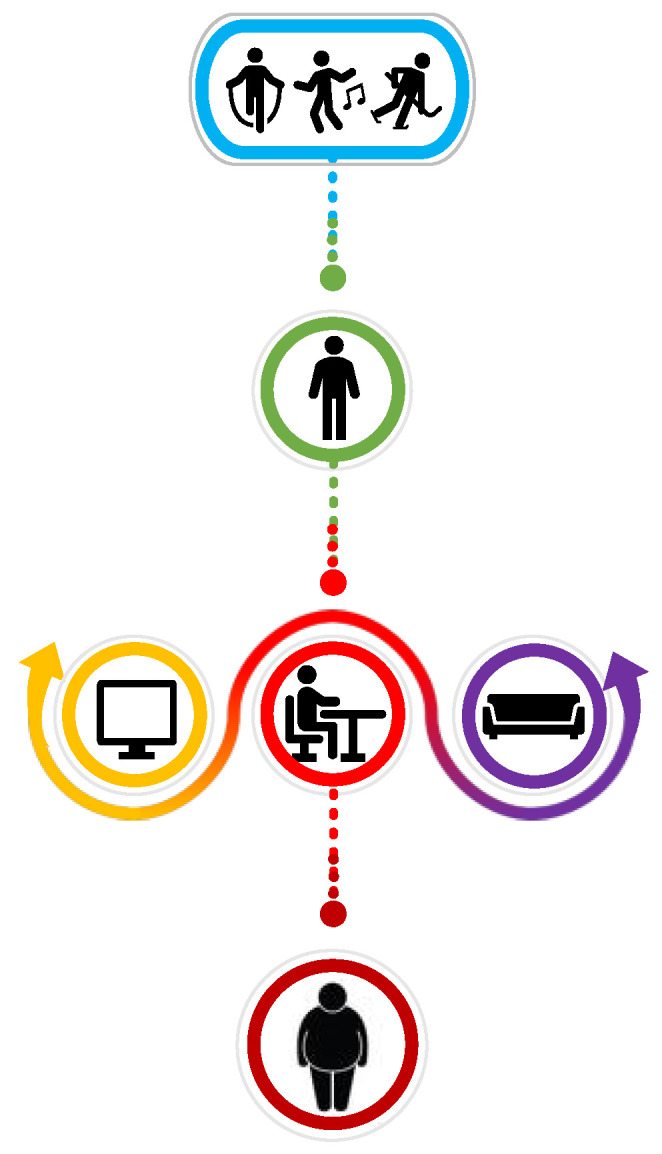
Screen time and physical inactivity as key factors that influence sedentary behavior in children with obesity.

Over the past few decades, the daily sedentary time of children and adolescents has increased at an unprecedented rate [86]. The dominant cause is the easy accessibility of screen-based activities, considered an engaging source of entertainment and a useful tool for forming and maintaining social connections [87]. Activities such as watching television and playing video games, computers and smartphones account for a substantial portion of total daily sedentary time [88]. Sedentary behavior in children and adolescents has been associated with reduced PA and the development of overweight status and obesity (Figure 2).

As reported, children who play video games more frequently have a higher weight [89,90] and an excessive caloric intake [91]. Children and adolescents engage in sedentary behavior for between 6 and 9 [92,93] and between 5 and 8 [94,95] hours a day, respectively, versus an average of only 12–13 min in vigorous activity [96]. Moreover, 79.9% of children spend at least some time playing video games; of these, 42.1% play exergames; therefore, it is important to consider that part of the time spent in front of the screen could be spent practicing PA [97].

Rather than competing against a highly valued activity, effective and accessible strategies are needed to encourage voluntary participation in daily PA in order to achieve recommended levels. Thus, the use of video games that promote activity can be an effective means of converting passive screen time into active time. The study of active video games (AVG) has shown that they involve an energy expenditure comparable to that of traditional activities such as swimming, walking, and jogging [86,87] and that the activity intensity can range from mild to intense [7]. Additionally, participation in exergames could increase the amount of other types of PA while reducing the total amount of time spent playing video games [16]. In the literature, there are heterogeneous studies on the effects exergames can have on PA and the improvement of sedentary time in children and adolescents who are overweight or obese [7,30,98]. The main inclusion criteria were age between 7 and 19 years and belonging to a BMI percentile above 85°. The duration of the intervention was a maximum of 40 weeks [24]; most studies proposed interventions lasting 10–12 weeks [16,25,26,27,28]. Sessions were self-regulated and took place mainly at the subjects’ homes, with the exception of one study which included sessions at a research laboratory [28]. Only Wagener et al. conducted a supervised program [26]. Control groups generally did not include any interventions; one included nonactive games [18], and another planned to carry out the intervention 10 weeks later [29]. The consoles used were mainly Kinect, Xbox 360, and PlayStation. The games proposed in the interventions were highly varied, for example, Dance Dance Revolution (DDR), Fitness Evolved 2012, Kinect Sport Season 2, and Disneyland Adventures.

As reported in Table 4, the main outcomes investigated in the studies are the sedentary time spent in front of the screen and the level of physical activity.

Of the studies investigated, eight showed increases, significant or not, in the level of PA practiced in the time and intensity parameters for the intervention group as compared to the control group [18,30,55,76,78,92,97]. Interestingly, in only three studies was this result associated with a reduction in sedentary time spent in front of the screen [18,30,97].

Some studies considered other parameters such as self-efficacy toward PA, self-esteem, and intrinsic motivation [18,93,97]. Table 4 highlights the significant and nonsignificant increases in the outcomes investigated, with the related tools.

Leaving subjects free to choose the type of exergames to use could increase these last parameters, together with entertainment [28]; it is logical to assume that this could increase playing time and therefore positively influence physical activity levels.

Furthermore, exergames can act as a gateway to promoting PA outside this context [28].

The results, although positive, are affected by some limitations. Almost all studies had undersized samples [16,25,26,27,28,29,30]; moreover, while there might have been significant differences between the intervention group and the control group, there was lack a stratification by age and gender [27]. The low validity of self-reported outcome measures (PA level) cannot guarantee objective data for PA monitoring [24,28], and the same is true for the evaluation of SST [29]. Short-term follow-ups do not ensure positive effects for a prolonged period of time. In fact, exergames can be an effective means of increasing the overall PA levels in children, but the importance of longer follow-ups to extend the long-term results is emphasized here again [16,25,26].

In light of this, studies are needed that include a larger sample and that test the sustainability and efficacy of exergames even after the end of the intervention [30].

A useful contribution could be made by experimenting with strategies and supports implemented to maintain the interest and enthusiasm of the game [31].

Overall, it can be said that technology-based interventions may be effective in increasing PA levels and reducing SST.

## 6. Conclusions

Nowadays, physical inactivity is the predominant cause for the increase in overweight and obese status in children and adolescents. Active video games (exergames), through increased physical activity, can help prevent overweight and obese status and its consequences in children and adolescents. In particular, active video games may have positive effects on the physical, psychological, and cognitive health of children and adolescents. Data from the literature indicate that exergames may exert a positive impact in childhood and adolescent obesity and may be considered a potential strategy for controlling weight gain and improving body composition by promoting PA and decreasing the time spent in sedentary behavior. Exergames are also an enjoyable form of PA for this population. Therefore, a prudent approach should be maintained, and additional high-quality research should be conducted to determine if exergames can be effectively used in the treatment of childhood obesity and if new digital media could be considered for combating sedentary behavior as supplementation of exercise rather than a replacement in educational programs for pediatric obesity prevention. Additionally, further research is also needed to assess exergames’ long-term effects.

## Figures and Tables

**Table 1:** Main characteristics of the investigated studies.

Author, Year	Study Design	Subjects (n°/Age)	Setting	Intervention Group (AVG/Frequency)	Control Group	Follow-Up
Bethea et al., 2012 [14]	Pilot study	IG: 34 CG: 28 9.9 ± 0.7 years	After school/Home	PlayStation 2^®^, DDR Extreme game^®^, dance mats up to 3 days/week for approximately 30 min during school and unlimited access at home	None	30 weeks
Murphy et al., 2009 [15]	RCT	IG: 23; 10.21 ± 1.67 years CG: 12; 7–12 years	Home	DDR 5 days per week	None	12 weeks
Ni Mhurchu et al., 2008 [16]	RCT	IG: 10 CG: 10 12 ± 1.5 years	Home	PlayStation Eye-Toy, and dance mat. Unspecified	Yes	12 weeks
Staiano et al., 2013 [11]	RCT	IG: 54 15–19 years CG: 16; 15–19 years	Home	Nintendo Wii Active exergame 30–60 min every school day during the lunch period or after school	Yes	12 weeks
Staiano et al., 2017 [17]	RCT	IG: 22 14–18 years CG: 19 14–18 years	Research laboratory	Dance exergaming Kinect for Xbox 360 (Microsoft Corporation, Redmond, WA, USA) 1 h of exergaming sessions per week	Yes	12 weeks
Trost et al., 2014 [18]	Randomized study	IG: 34; 10.1 ± 1.9 years CG: 41; 9.9 ± 1.5 years	Clinic	Kinect and Xbox 360 (Kinect sports) Unspecified	No active games	16 weeks
Maddison et al., 2011 [19]	RCT	IG: 160 10–14 years CG: 162 10–14 years	Home	PlayStation EyeToy (Sony), USB motion-capture camera, dance mat, and a selection of active video games (e.g., Play3, Kinetic, Sport, and Dance Factory; Sony) 60 min of moderate-to-vigorous physical activity on most days of the week	No active games	24 weeks
Baranowski et al., 2012 [20]	RCT	IG: 41 9–12 years CG: 43 9–12 years	Home	Wii console 30 min per day, 5 days per week	No active games	12 weeks
Gao et al., 2012 [21]	RCT	IG: 70 9–12 years CG: 33 9–12 years	Home	Dance Dance Revolution [DDR]; Konami Corporation Unspecified	No active games	8 weeks
Martínez-López et al., 2022 [22]	Quantitative longitudinal study	IG: 78 12–15 years CG: 86 12–15 years	Outdoor	Pokémon GO^®^; Unspecified	Yes	8 weeks

IG—interventional group; CG—control group.

**Table 2:** Investigated outcomes in the literature and the related tools used.

Author, Year	Outcome	Tool	Results	*p*-Value
Bethea et al., 2012 [14]	Physical activity/physical fitness/use of home Dance Dance Revolution/safety and acceptability/anthropometric/fasting metabolic profile at baseline, 12 weeks, and 30 weeks	FitnessGram^®^, a standardized physical fitness test battery.	IG were more active than CG	Significant
Murphy et al., 2009 [15]	Endothelial function and other risk factors in overweight children	Brachial artery flow-mediated dilatation (FMD), dual-energy X-ray absorptiometry, blood pressure measurement, fasting blood samples, insulin sensitivity index via oral glucose tolerance test, and physical fitness testing	IG had improved endothelial function and decreased body mass index, waist circumference, and systolic blood pressure than did CG	Significant
Ni Mhurchu et al., 2008 [16]	PA levels	Actigraph e PAQ-C	IG were more active than were CG	Significant
Staiano et al., 2013 [11]	Primary outcome: weight loss. Secondary outcomes: changes in BMI, self-esteem, and perceived physical competence.	Anthropometric measures, self-reported questionnaires, and physical fitness tests	IG had better weight loss and improved BMI, self-esteem, and perceived physical competence	Significant
Staiano et al., 2017 [17]	Body composition physical fitness, daily physical activity, sedentary time, dietary intake, and psychosocial well-being	Anthropometry, dual-energy X-ray absorptiometry, and magnetic resonance imaging to assess body composition. Youth Physical Activity Questionnaire, the Block Food Frequency Questionnaire, and the Pediatric Quality of Life Inventory	IG had significantly improved body composition, physical fitness, daily physical activity, and decreased sedentary time compared to CG. There were no significant differences in dietary intake or psychosocial well-being between IG and CG.	Significant
Trost et al., 2014 [18]	Sedentary behavior change	Actigraph	IG had decreases sedentary time compared to CG	Significant
Maddison et al., 2011 [19]	Changes in body composition (BMI, fat mass, waist circumference)	BIA, Actigraph	IG had decreased BMI, fat mass, and waist circumference compared to CG	Significant
Baranowski et al., 2012 [20]	Physical activity level	Actigraph GT33 x accelerometers	IG were not more active in general, or at any time, than were CG	Nonsignificant
Gao et al., 2012 [21]	Physical activity level	Actigraph accelerometer and AVG performance scores	IG had higher physical activity levels than did CG	Significant
Martínez-López et al., 2021 [23]	Physical fitness and fatness	Measures of physical fitness (CRF, S/A, MS) were assessed using the ALPHA health-related fitness test battery for youth. Measures of fatness were BMI (assessed through height, measured by ASIMED^®^ B-type-class III (Guayaquil, Ecuador), and weight, measured through a portable height meter SECA 214^®^ Ltd., Reinach, Switzerland), BIA (measured through Biospace InBody 720 bioelectrical impedance analyzer) and waist and hip circumferences (measured with tape measure)	IG had greater improvement for CRF, BMI, and %BF than did CG	Physical fitness: significant for CRF but not significant for S/A or MS; Fatness: significant for BMI and %BF but not significant for WHI

IG—interventional group; CG—control group; PA—physical activity; BMI—body mass index; BIA—bioelectrical impedance analysis; CRF—cardiorespiratory fitness; S/A—speed/agility; MS—muscular strength; %BF—percentage of body fat; WHI—waist–hip index.

**Table 3:** Main characteristics of the studies investigated.

Author, Year	Study Design	Subjects (n°/Age)	Setting	Intervention Group (AVG/Frequency)	Control Group	Follow-Up
Ni Mhurchu et al., 2008 [16]	RCT	IG: 10 CG: 10 12 ± 1.5 years	Home	PlayStation Eye-Toy, and dance mat. Unspecified	No active games	12 weeks
Simons et al., 2015 [24]	RCT	IG: 134; 13.7 ± 1.3 years CG: 126; 14.1 ± 1.3 years	Home	PlayStation Move (Sport Champions, Move Fitness, Start the Party, Medieval Moves, Dance Star Party and Sorcery) At least one hour a week	None	40 weeks
Graves et al., 2010 [25]	RCT	IG: 22 CG:29 9.2 ± 0.5 years	Home	PlayStation 2 and 3 and Nintendo Wii; jOG packing Unspecified	None	12 weeks
Wagener et al., 2012 [26]	RCT	IG: 21 CG: 20 12–18 years	Clinic	Exergames based on supervised group dance. 3 times a week; 2 × 15’ for the first session; 10’ rest between sets 4 × 15’ for subsequent sessions; 5’ rest between sets	None	10 weeks
Maloney et al., 2012 [27]	RCT	IG: 33; 12.9 ± 2.36 years CG: 31; 11.73 ± 2.38 years	Home	PlayStation 2 (DDR). Average of 89’ per week	None	12 weeks
Staiano et al., 2017 [28]	Original article	IG: 19 girls; 15.3 ± 1.3 years CG: 18 girls; 16.1 ± 1.3 years	Research laboratory	Dance exergaming Kinect for Xbox 360 (Microsoft Corporation, Redmond, WA, USA) Thirty-six 60 min sessions	None	12 weeks
Trost et al., 2014 [18]	Randomized study	IG: 34; 10.1 ± 1.9 years CG: 41; 9.9 ± 1.5 years	Clinic	Kinect and Xbox 360 (Kinect ports) Unspecified	No active games	16 weeks
Maloney et al., 2008 [29]	Pilot study	IG: 40; 7.5 ± 0.5 years CG: 20; 7.6 ± 0.5 years	Home	PlayStation2 game console DDR MAX2 game (Konami of America, Redwood City, CA, USA), 120 min per week of DDR, preferably divided over four sessions	Wait-list control (10-week delay)	28 weeks
Staiano et al., 2018 [30]	RCT	IG: 23 CG: 23 11.2 ± 0.8 years	Home	Kinect and Xbox 360 (Your Shape: Fitness Evolved 2012, Just Dance 3, Disneyland Adventures, and Kinect Sports Season 2) One hour per session, 3 times a week + weekly/bi-weekly video chat sessions with an athletic trainer	None	24 weeks
Liang et al., 2020 [31]	RCT	IG: 30 CG: 57 10.5 ± 0.8 years	School	Xbox 360 Kinect Two 1-h sessions per week.	None	8 weeks
Christison et al., 2012 [32]	Prospective observational pilot study	IG: 48 8–16 years	Home	DDR, exerbike XG, Nintendo Wii 10 weekly 1 h facilitated activity sessions: 5 one-hour exergaming sessions; 5 one-hour combined exergaming/traditional exercise sessions	None	18 months

IG—interventional group; CG—control group; PA—physical activity.

**Table 4:** Outcomes investigated in the literature and the related tools used.

Author, Year	Outcome	Tool	Results	*p*-Value
Ni Mhurchu et al., 2008 [16]	PA levels	Actigraph e Paq-c	IG more active than CG	Significant
Simons et al., 2015 [24]	Energy expenditure	Actigraph	IG > CG	Nonsignificant
Graves et al., 2010 [25]	Perceived competence to exercise/relations with parents/self-esteem	Perceived Competence Scale (PCS)/Parent Rating Scales-Adolescent version (PRS-A)/Adolescent Self-Report Scales (SRP-A)	IG had an increase in perceived competence and relationships with parents	Significant
Wagener et al., 2012 [26]	Screen time	Actigraph	IG < CG	Significant
Maloney et al., 2012 [27]	Total sedentary screen time/self-reported nonactive videogame time	Flemish Physical Activity Computerized Questionnaire (FPACQ)	IG had a reduction in screen time and passive video game participation	Significant
Staiano et al., 2017 [28]	PA levels	Actigraph	IG had improved self-efficacy during intervention compared to GC	Non Significant
Trost et al., 2014 [18]	Sedentary behavior change	Actigraph	IG had decrease sedentary time compared to GC	Significant
Maloney et al., 2008 [29]	Screen time/self-esteem/PA time	Self-perception profile for children (SPPC)/healthy style questionnaire	IG had reduced screen time and increased motor activity hours and self-esteem	Significant
Staiano et al., 2018 [30]	PA levels	Actigraph	IG had increased levels of moderate-to-vigorous motor activity compared with CG	Significant
Liang et al., 2020 [31]	PA/PA during leisure time/self-efficacy toward PA/screen time/intrinsic motivation	Accelerometry (ActiGraph GT3X+; ActiGraph Inc., FortWalton Beach, FL, USA)/Godin-Shephard Leisure-Time Physical Activity Questionnaire/13-item self-report survey/NHANES/IMI	IG self-reported an increase in PA (*p* = 0.035) and fewer hours watching television or videos (*p* = 0.01) after the intervention; significantly improved self-efficacy toward PA (*p* = 0.028); and highly rated intrinsic motivation toward exergaming.	Significant
Christison et al., 2012 [32]	PA/SST (sedentary screen time)	ActiGraph accelerometer (MTI Health Systems, Ft. Walton Beach, FL, USA)/jointly self-reported measure	Increase in vigorous PA and reduction in light PA/decrease in SST of –1.2 ± 3.7 h per week (hpw)	Significant

IG—interventional group; CG—control group; PA—physical activity.

## Data Availability

Not applicable.
